# An empirical investigation of the efficiency effects of integrated care models in Switzerland

**DOI:** 10.5334/ijic.685

**Published:** 2012-01-13

**Authors:** Oliver Reich, Roland Rapold, Magdalena Flatscher-Thöni

**Affiliations:** Department of Public Health and Health Technology Assessment, UMIT, University of Health Sciences, Medical Informatics and Technology, Eduard Wallnöfer Zentrum 1, A-6060 Hall in Tyrol, Austria; Division of Health Economics and Health Policy, Helsana, Zürichstrasse 130, CH-8600 Dübendorf, Switzerland; Department of Public Health and Health Technology Assessment, UMIT, University of Health Sciences, Medical Informatics and Technology, Eduard Wallnöfer Zentrum 1, A-6060 Hall in Tyrol, Austria

**Keywords:** Switzerland, integrated care, efficiency effects, selection effects

## Abstract

**Introduction:**

This study investigates the efficiency gains of integrated care models in Switzerland, since these models are regarded as cost containment options in national social health insurance. These plans generate much lower average health care expenditure than the basic insurance plan. The question is, however, to what extent these total savings are due to the effects of selection and efficiency.

**Methods:**

The empirical analysis is based on data from 399,274 Swiss residents that constantly had compulsory health insurance with the Helsana Group, the largest health insurer in Switzerland, covering the years 2006–2009. In order to evaluate the efficiency of the different integrated care models, we apply an econometric approach with a mixed-effects model.

**Results:**

Our estimations indicate that the efficiency effects of integrated care models on health care expenditure are significant. However, the different insurance plans vary, revealing the following efficiency gains per model: contracted capitated model 21.2%, contracted non-capitated model 15.5% and telemedicine model 3.7%. The remaining 8.5%, 5.6% and 22.5%, respectively, of the variation in total health care expenditure can be attributed to the effects of selection.

**Conclusions:**

Integrated care models have the potential to improve care for patients with chronic diseases and concurrently have a positive impact on health care expenditure. We suggest policy-makers improve the incentives for patients with chronic diseases within the existing regulations providing further potential for cost-efficiency of medical care.

## Introduction

A high increase in health care expenditure (HCE) can be observed worldwide. With expenditure of 4810 US$ PPP^1^ in 2009, Switzerland showed one of the highest HCE per capita in the world after the US [1]. Factors, such as technological change and population expectations are pushing up health spending and will continue to drive costs even higher in the future [2]. The development of health care expenditure in Switzerland is estimated for 2009 to amount to 61 billion Swiss francs and in 2010 to exceed 62.5 billion Swiss francs [3]. This represents approximately 11.5% of gross domestic product (GDP) vs. 2 billion francs in 1960, with a share of around 5% of GDP. This growth corresponds to an annual increase of roughly 7% in the last 50 years [1].

However, compared with other countries, the health care system in Switzerland is not only expensive, but also provides good to very good quality outcomes [4–6]. Higher health spending is reflected in the improvement of objective health indicators, such as life expectancy at birth. In Switzerland in 2008, this outcome indicator was 82.2 years, which is behind Japan and is considered to be the second best value of the 30 OECD countries [1]. Given the high health care expenditure of the Swiss system, together with the fact that other countries have a similarly good system with significantly lower costs, the efficiency of the Swiss system and thus also necessary reforms can be put into question [5].

The aforementioned OECD analysis of the Swiss health system brought to the surface the fault that medical provision is basically too fragmented. There is a lack of co-ordination. Overuse and misuse are often the result. Here Integrated Care^2^ (IC) may remedy the situation by linking providers to integrated networks. By vertically integrating health insurance and health care provision, IC may improve the allocation of resources in health care while limiting HCE. But what is integration and can one define integrated care? A good general definition of integrated care has been established by Kodner and Spreeuwenberg: “Integration is a coherent set of methods and models on the funding, administrative, organisational, service delivery and clinical levels designed to create connectivity, alignment and collaboration within and between the cure and care sectors. The goal of these methods and models is to enhance quality of care and quality of life, consumer satisfaction and system efficiency for patients with complex, long-term problems cutting across multiple services, providers and settings. The result of such multi-pronged efforts to promote integration for the benefit of these special patient groups is called ‘integrated care.’”[7]. In Switzerland various methods and models in the area of IC can be found [8]. On the one hand integrated care models (ICM) and on the other hand various different methods and instruments, such as disease management [9], case management, gatekeeping, demand management, chronic care management or guidelines. Very often these integration methods and instruments are embedded and utilised in ICM. Our study focuses solely on the effects of integrated care models in Switzerland.

Social health insurance (SHI) is compulsory for the population in Switzerland. Basic insurance allows the insured person the freedom of choice of doctors in the outpatient sector and unlimited access to general practitioners and specialist physicians. Alternative forms of insurance exist with the option of restrained choice of medical providers granting policyholders discounts on the basic premium if they agree to sign up to integrated care models and only consult certain providers. The selected doctor or medical call centre incorporated in such an ICM acts as a family doctor, primary physician or gatekeeper. He covers all primary care and co-ordinates, if necessary, any further referrals to specialists or hospitals. Treatment and consultations without prior referral by the primary physician or telemedicine service are usually not covered by the health plan.

**Figure 1.**
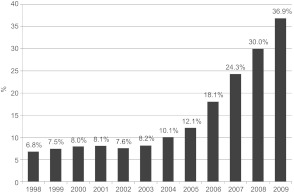
Proportion of population in an integrated care model per annum.

Switzerland has vast and lengthy experience within Europe in the development of integrated care models [10] and carries a wide range of insurance plans. In 2009 nearly 37% of the Swiss population enrolled in these models, compared with a rate of 6.8% in 1998 [11]. Figure 1 presents the development from 1998 to 2009. This percentage would probably be even higher if the health insurance plans were to offer ICM in all cantons, regions and cities.

The main forms of integrated care models in Switzerland can be characterised as follows:

### Contract models with capitation

In these models a group of providers incorporate a network, which then makes a co-operation agreement with a health insurer. These networks can consist of a group of independent individual (general) practitioners or a Health Maintenance Organisation (HMO). Within the context of this contract, the network takes over collective responsibility for a financial budget. The remuneration by capitation gives the provider-network a calculated flat per capita allowance, for which it provides health care for the signed-up insured persons for a certain period. For this reason these models are also called per capita funding models. However, the financial budget is in practice merely a virtual cost target (virtual budget) and not a payment per se (disbursed budget). Providers do not receive a fixed periodic payment for each patient and are paid by the nationwide fee-for-service scheme. If the virtual budget is undershot at the end of the year, the difference is divided between the provider network and the health insurance plan according to the previously contractual agreed distribution. Therefore, the provider network will benefit from this participation. On the other hand, if the virtual budget is exceeded, the negative difference is shared accordingly. The provider network is required to pay its part of the difference to the insurer. Thus, Swiss integrated care models under capitation payment differ from health plans found abroad [12].

Nevertheless, the importance of the capitation lies probably not only in the financial incentive, but in the redistribution of responsibilities. This funding model merges medical and economic responsibility. Since economic responsibility includes the entire benefit of medical services^3^, the network also assumes responsibility for the whole treatment process, particularly interface management between different institutions and specialities. This potentially leads away from volume based-medicine to more patient focused-care, taking economic aspects into account. Of the nationwide 37% of policyholders who have selected an IC model, approximately 4% are in a capitated ICM (year 2009) [13].

### Contract models without capitation

The attributes of these models are identical to the above, except that the provider networks are not paid capitation. In addition to the aforementioned HMOs and the family doctor models, this category covers a new model variation: medical call centre or telemedicine models. In Switzerland there are several call centres that focus on the provision of medical advice. The aim of this offer is optimal supply of medical services for the patients signed up. This is done by medical professionals answering patient questions with computer assistance and referring the patients in need of a doctor or a hospital. As in all ICM, the insured are obliged to consult their chosen gatekeeper, in this case the medical call centre, before turning to other medical providers. Approximately 21.4% of the insured have enrolled in this category of contract models without capitation, whereas 6.5% are in family doctor (B1) models and 14.9% in medical call centre models (B2)^4^ [13].

### Non-contracted models

Unlike both the above-mentioned models, the providers in the so-called list model are not contractually incorporated into a health insurance plan. These models are only insurance products, where the health insurer unilaterally defines lists of selectable medical service providers as gatekeepers. Often these list models are offered in regions without existing cooperation agreements with networks. Health plans utilise this form of ICM because of the possibility of applying premium rebates, which are naturally very popular with customers. Approximately 11.6% of the insured have opted for this health plan form^5^. Insured persons enrolled in these non-contract models are excluded from our analysis in the present study. This is owing to the obviously extremely low co-ordination and integration impact in the described non-contracted models on health care provision.

The raw data show that the health expenditure of those subject to an ICM is 1675 Swiss francs is less than half that of those who remained with their original plan (4022 Swiss francs) [11]. However, the question arises, as to what extent these spending differences are due to the efficiency of integrated care models and whether they are moreover based on potential selection effects. Van de Ven et al. [14] already indicated the evidence of increasing risk selection tendencies in Switzerland in the area of integrated care, as in health insurers applying ICM-options, in order to attract good risks. They, therefore, emphasised the importance of a good risk adjustment scheme in a competitive insurance market. This paper looks at the impact of integrated care models on health care expenditure in Switzerland, while simultaneously accounting for selection effects and, therefore, makes a contribution to the discussion on possible expenditure containment measures.

The paper is structured as follows. *Background* section 2 starts with a short review of the literature. *Data and method* section 3 contains the data and descriptive statistics, as well as the method to estimate the efficiency effect of integrated care models on HCE. Results are shown and discussed in *Empirical results* section 4, while the last section ends with our conclusions.

## Background

The objective of this paper is to investigate the efficiency gains of integrated care models in Switzerland. A comprehensive review of the field of empirical literature most closely related to the question of interest is provided for international research studies in Glied [12] and specifically for Switzerland in Berchtold and Hess [8, 15]. Generally, existing studies found that HMO-types of integrated care models lower health care expenditure. However, as Glied [12] points out, the problem of selection into integrated care model health plans is of a great importance when focusing on the impact on HCE. Therefore research studies have to account for selection effects in order not to overestimate the effect of gatekeeping and other integrated care instruments through other unobserved variables.

To our knowledge, only four studies have previously analysed this field in Switzerland using individual data. Lehmann and Zweifel [16] estimated the efficiencies of a HMO, family doctor model and a list model using panel data from a Swiss health insurer. They found 62% for the HMOs, 34% for the family doctor model and 39% for the list model, lower health care expenditure than for traditional plans. These cost savings are based, however, on evidence found for risk selection effects, which accounted for one-third of the cost advantage in the case of HMOs and less than half for the list model. The remaining substantial efficiency gain was assigned to innovation effects. Schwenkglenks et al. [17] assessed the economic efficiency of a family doctor plan compared with a fee-for-service plan in the region of Aarau, Switzerland. The study population was based on a random selection from insurance enrolment files and finally counted 466 persons. The estimated results after the multivariate adjustment revealed cost savings of 15–19% due to the family doctor model. Beck [18] intensely studied the effect of managed care on the level of HCE by applying a matching technique, in order to estimate the efficiency gains on policyholders of a major Swiss health insurer. The study covered the years of 2006 and 2007 and all 18 insurance models with a capitation agreement, and corrected the morbidity discrepancies between the insurance collectives. Despite the variation of the results among different models, the investigated managed care plans were found to reduce health care expenditure on average by 8.7%. The best result was achieved by one of the models at 18.5%, the worst model leading to a (statistically insignificant) negative effect of –3%. Finally, Grandchamp and Gardiol [19] used a two-step model approach to estimate empirically the efficiency of a Swiss telemedicine service using claims’ data from a Swiss health insurer covering 160,000 insured adults. They compared the health expenditure of insured persons in a telemedicine service model with those assigned to the conventional insurance plan. About 90% of the difference in health expenditure can be explained by selection and incentive effects. The remaining 10% of savings due to the efficiency of the telemedicine service amount to about 150 Swiss francs per year per insured.

The innovation to the preceding studies is that we analyse empirically the impact of ICM over a longer period of time as well as performing this for all contract-based models, meaning not only on capitated integrated care or telemedicine service models but also on non-capitated ICM. Furthermore, we take up the request for a simpler method from Beck [18] and present an alternative methodical approach.

## Data and method

Our analysis is based on data of the health care insurance group, Helsana, the largest health insurer in Switzerland, including 1.37 million individuals with compulsory health insurance. As insurance switchers would introduce a possible bias in the efficiency estimation, the utilised data sample comprises 399,274 Swiss residents that constantly had compulsory health insurance within the same insurance plan, covering the years 2006–2009, i.e. a total of 1,597,096 observations. For each individual, we observe the annual amount of health care expenditure for the period in question. Furthermore, since our main objective is to investigate the efficiency impact of ICM over time, we incorporated only individuals with health care expenditure during this period. Hence, individuals enrolled in a telemedicine call centre model who only addressed the call centre without any further health consultations were also not included in this investigation. This aspect is a potential source of bias and may lead to an underestimation of the efficiency effect in the telemedicine model.

It is reasonable to assume that this sample is highly reliable, since the administrative claims data collected by the insurer cover nearly all health care invoices. The Helsana Group offers the basic compulsory insurance health plan as well as all afore-mentioned types of integrated care models. Therefore, the models in this study were divided into four groups: basic compulsory insurance plan and contracted ICM, which has three categories: A) capitation model, B1) family doctor model, and B2) telemedicine model. The ICM-share of the total insured has increased constantly over the period in question from 13.7% in 2006 to 30.6% in 2009. However, the variation between the various integrated care models is vast. The most popular model is the B2) telemedicine model with a share of 18.9% of total insured in 2009, mainly owing to the fact that, in contrast to the other models, this model can be offered in all Swiss regions.

**Figure 2.**
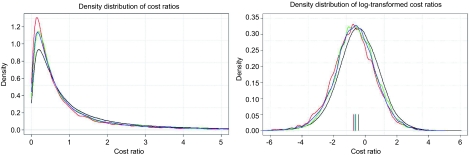
Distribution of cost ratio per model (left) and distribution of logarithmic cost ratio per model (right) Black: Basic compulsory insurance; red: A) capitation models; green: B1) family doctor model; blue: B2) telemedicine model.

In order to evaluate the efficiency of the different integrated care models, we apply an econometric approach. We specify the following empirical model for the dependant variable cost ratio of total costs per individual *i* in year *t*, simultaneously summarising the independent variables:


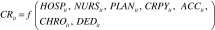


where:

CR: Cost ratio total costs: the total health expenditure of the individual in Swiss francs divided by the average cantonal health expenditure according to the Swiss risk equalisation scheme^6^. The distribution of the cost ratio for each insurance model is shown in Figure 2. The corresponding highlighted colours in Figure 2 are defined as follows: black, basic compulsory insurance; red, A) capitation models; green, B1) family doctor model and blue, B2) telemedicine model. The distribution of the ratio spread is askew (left plot), whereas the distribution of a logarithmic ratio (right plot) can well approximate a Gaussian distribution
HOSP: Hospital stay: dummy variable equal to 1 if insured showed a hospital stay of more than 2 days in the preceding year and zero otherwise
NURS: Nursing home stay: dummy variable equal to 1 if insured showed a nursing home stay of more than 2 days in the preceding year and zero otherwise
PLAN: Type of health plan: we defined three dummy variables according to the type of health plan chosen by member, whereas the compulsory basic insurance plan is the according reference value:
  Capitation model=CAP: dummy variable equal to 1 if insured is a member of a capitation model and zero otherwise;
  Family doctor model=FDM: dummy variable equal to 1 if insured is a member of a family doctor model and zero otherwise;
  Telemedicine model=TEL: dummy variable equal to 1 if insured is a member of a telemedicine model and zero otherwise
CRPY: Cost ratio preceding year: the total health expenditure of the individual in Swiss francs, divided by the average cantonal health expenditure according to the Swiss risk equalisation scheme of the preceding year. These ratios are not used directly, but categorised into 24 different groups of cost ratios. The first 20 groups are formed with approximately the same number of insured persons per group (4.8% per group). The group with the highest cost ratios is then further divided into 4 last groups in order to be able to handle the very high cost ratios more accurately. The cost ratios are ultimately divided into 24 groups, leading to 23 corresponding Dummy variables, whereas the first cost ratio group is the according reference value.
  In applying this variable to the included integrated care models, we have to account for the assumption that the costs of the insured persons enrolled in these models are lower than the basic insurance scheme. Therefore, the corresponding costs of the preceding year need to be increased by the efficiency effect achieved to the level of the basic insurance scheme. Without applying this correction, the efficiency effect of integrated care models will result to be too high. We describe the repeated process used further below.
ACC: Accident coverage: dummy variable equal to 1 if insured possessed additional accident insurance cover and zero otherwise
CHRO: Patients with a serious chronic disease such as diabetes, cardiac disorders or rheumatism have a pattern of high recurring costs over time. We assume that patients with a chronic illness can be identified by capturing the number of quarters within a year, in which claims were billed over the threshold of 500 Swiss francs. An insured person falls into our definition of chronic disease if the number of quarters is > 0. We therefore defined four dummy variables according to the number of quarters over the mentioned threshold, whereas no quarters is the corresponding reference value:
  1 quarter=CHRO1: dummy variable equal to 1 if insured has 1 quarter within a year over threshold and zero otherwise;
  2 quarters=CHRO2: dummy variable equal to 1 if insured has 2 quarters within a year over threshold and zero otherwise;
  3 quarters=CHRO3: dummy variable equal to 1 if insured has 3 quarters within a year over threshold and zero otherwise;
  4 quarters=CHRO4: dummy variable equal to 1 if insured has 4 quarters within a year over threshold and zero otherwise
DED: Deductible class: dummy variable equal to 1 if insured chose a deductible higher than Swiss francs 500 and zero otherwise

The estimation of equation 1 requires the specification of a functional form. By applying this form, the model can be written as:


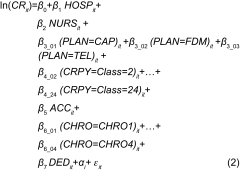


To choose the econometric approach, we consider the fact that the independent variables regarding the type of health plan (CAP, FDM and TEL) are of central substantive interest in the present study. The data of the year 2009 were used to develop the linear regression model with the variables shown. By reason of the fact that multiple records of data per person are present in this analysis, a linear regression is not feasible. To account for the different levels of cost ratios for the different individuals, the linear regression model, using fixed-effects, is augmented with a random-effect αi. The resulting mixed-effects model was calculated with the nlme package of R [www.r-project.org], proposed by Pinheiro and Bates [20]. The residual unexplained error term in linear regression corresponding to e_it_, is in this mixed-effects model divided into the random-effect a_i_ (between group/person error) and the remaining residual error term e_it_ (within group/person error).

The predictor variables presented are the successful subset of an earlier and larger set of predictors estimated. The regression model building and covariate selection was performed step by step. Further candidates assessed were premium region, pharmacy cost groups and various interactions between covariates. In terms of the CHRO variable utilised, we also examined different thresholds. Apart from the 500 Swiss francs, a 2500 Swiss francs threshold was applied, plus various possible grouping options. Our final model was established from different combinations of predictor variables due to their significance and by applying likelihood ratio statistics.

According to prior research [21], the most essential and significant explanatory variable for health care expenditure estimations are the costs of the preceding year (CRPY). We applied this finding in our analysis at hand. As we already mentioned in the variable description of CRPY, we have to increase the corresponding costs of the preceding year by the achieved efficiency effect to the level of the basic insurance scheme. Without applying this correction, the efficiency effect of integrated care models will result to be too high. One advantage of this study is that we base our analysis on four consecutive years and estimate a continuous efficiency effect over the whole period. This calculation of the efficiency effects requires a repeated procedure. First of all, we corrected the CRPY for individuals included in integrated care models with an anticipated entry value, differing according to the model type, and then estimated the efficiency effect. Next, the achieved efficiency effect estimations were inserted as correction values for the costs of the preceding year and afterwards a new estimation was performed. This procedure was continued until the model estimations were equivalent to the applied corrections made to CRPY. We tested different entry values for the corrections. Finally, they all led to the identical estimations for the efficiency effect and therefore we assume possession of a robust result.

Descriptive statistics for the sample are presented in Table 1. As can be seen and as expected, the cost ratios of all integrated care models were lower than in the sample covering the basic compulsory insurance model. The total effect of cost savings per model in comparison to the basic insurance scheme is –29.7% for the contracted models with capitation, –21.1% for Family doctor models and –22.5% for telemedicine models.

In order to derive a further explanation of the efficiency effects in integrated care models, we shall also compare the cost ratios of the four most frequent pharmacy cost groups (PCG) between the four different insurance plans. According to Lamers and van Vliet [22], PCGs identify patients with a serious chronic illness by means of drug claims submitted for the corresponding medicines prescribed. Our Swiss definition of the utilised pharmacy cost groups is based on Beck [23] and distinguishes between 13 different groups^7^.

## Empirical results

The aim of this paper is to address the potential of efficiency effects in integrated care models in comparison with the basic compulsory insurance scheme in Switzerland. The handling of selection effects within the models is controlled in our econometric approach. Estimation results from mixed-effects model of equation 2 are presented in Table 2. The estimated sampling correlations among the fixed-effect coefficient estimates were analysed. The corresponding correlations revealed very low levels among the coefficients to the central integrated care model variables (CAP, FDM and TEL) and, therefore, no further bias in this area can be stipulated.

Our results for the efficiency effects indicate that contracted types of integrated care models in Switzerland are significantly associated with lower health expenditure. However, the different insurance plans vary, revealing the following efficiency gains per model: contracted capitated model 21.2%, contracted non-capitated model 15.5% and telemedicine model 3.7%. In terms of selection effects in the different models, the residual of the overall average cost savings mentioned in *Data and method* section 3 less the efficiency gains can be calculated. Table 3 illustrates the different effects per model.

These findings are consistent with previous studies, which have shown that health care costs are lower in integrated than in non-integrated systems [16–19, 24]. However, apart from the telemedicine model, the impact of the stated efficiency effects in our investigation differentiates from prior studies. The total efficiency cost advantage is nearly exactly quantified between the values by Lehmann and Zweifel [16] and Beck [18] for the corresponding capitated models. The econometric analysis performed by Lehmann provided high efficiency gain levels of –40% for the capitated models. Yet, the analysed insurance plan differs from the one in our study since it was based on HMOs that were operated and funded by a health insurer. This situation might lead to a more restrictive care treatment within the insurance-held own HMO and therefore to higher cost savings. Beck described the efficiency effects only of capitated models over 2 years and presented cost advantages of 8.7% and selection effects of about 52%. In contrast to the findings of Beck, our selection effects play a less dominant role. Our estimations show selection effects of 8.5% on average in comparison to non-ICM-insured. The study design differs, however, to the present analysis and may be be put forward to explain the difference. In his study, efficiency gains are calculated for 18 individual physician networks separately instead of for the insurance plan type as a whole. The corresponding results between the networks vary greatly from –3.7% to –18.5%^8^. Other types of integrated care models were not analysed by Beck. In terms of the efficiency effect found for family doctor models (–15.5%) the expected result is perfectly in line with the effect of –15 to –19% identified by Schwenkglenks et al. [17] and more or less comparable with the study performed by Lehmann with efficiency gains of –10%. The quantified efficiency effect for this type of integrated care model seems to be rather consistent over the years. His study covered the years from 1997 to 2000. The present investigation covers the years from 2006 to 2009 and we presume that there has been some kind of development in the area of family doctor models within the last decade. Expected positive results were found with the effects generated by telemedicine models. The efficiency effect (3.7%) is consistent with prior findings by Grandchamp and Gardiol [19].

The central instrument of the current integrated care models in Switzerland is gatekeeping. The gatekeeper tries to coordinate the care of patients among multiple providers in order to avoid wasteful duplication of diagnostic testing, perilous poly-pharmacy and confusion over conflicting care plans. A potential reason for the stated efficiency effects in integrated care models may be due to better co-ordination and integration of medical provision leading to a more effective and efficient delivery of care. Previous research reveals the importance of care co-ordination [8, 15, 25–27] and indicates the potential for continuous cost containment by applying integrated care models. It also concurrently indicates with these, the corresponding instruments in the health care system. A comparison of the cost ratios of the four most frequent pharmacy cost groups (PCG) between the four different insurance plans can be put forward in order to explain the efficiency effects achieved in integrated care models. Table 4 presents the corresponding values per insurance model. The results corroborate the assumption that integrated care models can exert a positive financial impact by a better co-ordination of medical care, especially in the area of chronic diseases. The cost ratio of common chronic diseases, such as asthma and chronic obstructive pulmonary disease in capitated models are treated 31% more cost-efficient than the same chronic patient group in the basic compulsory insurance scheme.

However, apart from the aforementioned effects on health expenditure, integrated forms of practice also provide higher quality of care. The findings of previous research display that physician group type influences health care quality considerably [28–31]. Therefore, in summary, as we look for more efficient health care delivery and better quality of care, it may be worthwhile to look at integrated care models as an example of an efficient and high quality model in Switzerland.

Turning to the remaining exogenous factors, the regression analyses also show that they are statistically significant at the 99% confidence level. This indicates that these variables should be taken into account when conducting future research in this area. The effect on the dependent variable cost ratio differs, however, to great extent. Variables with a very strong influence are chronic patients, hospital last year and cost ratio in the previous year. A surprisingly small impact is exerted by the variable deductible class.

This study has several strengths. To our knowledge and contrary to other studies, it is the first empirical investigation to have applied a uniformed analysis for all types of contracted integrated care models found in Switzerland. This guarantees a uniform data set and presents an ideal comparison for the estimation of potential efficiency effects in these types of insurance plans. In addition, our analysis is based on a period of four consecutive years, which allows us to capture the long-term efficiency effect and to reduce possible bias’ due to individual changes of insurance models or changes of social health insurer within the period.

We also want to point out a limitation of our study. From previous unpublished research^9^, we estimate that 2–3% of all claims invoices are paid directly by the patient (e.g. due to high deductibles chosen) and not reimbursed by the health insurer. This may lead to a possible bias due to a mixture of the different effects in the estimation and missing claims data.

## Conclusion

This empirical study reaffirms that integrated care models can contribute effectively and recurring as a cost containment measure in the Swiss health care system. Our attempt to investigate the differences in health care expenditure due to efficiency effects rather than mere risk selection was based on a panel dataset from a major Swiss health insurer with 399,274 insured individuals, covering the years 2006–2009. By controlling the effects of risk selection the average gross reduction in costs achieved by capitated integrated care models, such as HMO, attain 21.2% in comparison with the basic insurance scheme. Family doctor models without capitation financing and the telemedicine models show lower cost savings of 15.5% and 3.7%, respectively. These results are robust and are consistent with previous research findings in Switzerland. However, we must stress that the aforementioned efficiency effects represent an average figure per model. For example, 25 different network providers are included within the capitation models and the variation between the network providers can be assumed to be vast. Nevertheless, against the background of our results, we suggest social health insurance companies increase their integrated care activities in order to secure the benefits of a competitive health insurance market beyond risk selection. Given the upcoming challenges, especially in terms of increasing chronic diseases combined with demographic ageing, the management of chronic diseases is high on the agenda. Integrated care models have the potential to improve care for patients with chronic diseases and concurrently have a positive impact on health care expenditure. We suggest that health care decision-makers and policy-makers improve the incentives for patients with chronic illnesses within the existing regulations. However, as prior research has revealed, the financial incentives for patients must be substantial [32]. This might lead to a higher proportion of chronic patients enlisting in integrated care models providing further potential for cost-efficiency of medical care owing to enhanced co-ordination within the corresponding provider networks of integrated care.

## Figures and Tables

**Table 1.** Sample characteristics on variables

	Basic compulsory insurance model with free choice of provider	A) Contracted models with capitation	B) Contracted models without capitation	
			B1) Family doctor model	B2) Telemedicine model
*i*	1,430,704	20,336	45,976	100,080
*t*	2006–2009	2006–2009	2006–2009	2006–2009
N	357,676	5084	11,494	25,020
*Cost ratio total costs mean*	1.535	1.079	1.211	1.190
Std. dev.	3.737	2.265	3.038	2.706
*Age groups*				
0–18 years	12.60%	6.88%	6.95%	10.77%
19–25 years	4.52%	3.63%	4.14%	4.90%
26–30 years	2.75%	2.67%	1.57%	2.72%
31–35 years	3.45%	3.57%	1.57%	3.54%
36–40 years	4.33%	4.89%	2.44%	4.61%
41–45 years	5.54%	6.32%	4.29%	5.92%
46–50 years	6.14%	7.14%	5.69%	6.75%
51–55 years	6.96%	6.90%	6.93%	8.31%
56–60 years	8.31%	7.34%	8.57%	10.20%
61–65 years	9.20%	8.78%	9.97%	11.62%
66–70 years	8.60%	8.90%	10.75%	9.45%
71–75 years	8.75%	10.68%	11.96%	8.43%
76–80 years	8.15%	10.58%	11.23%	6.39%
81–85 years	6.20%	7.47%	8.50%	4.20%
86–90 years	3.24%	3.25%	4.02%	1.69%
91-years	1.26%	0.99%	1.42%	0.49%
*Deductible class* (>500 Swiss francs)	9.76%	17.89%	10.24%	28.60%
*Hospital stay*	13.01%	11.28%	13.00%	10.69%
*Nursing home stay*	3.05%	1.79%	3.48%	1.09%
*Accident coverage*	23.25%	29.22%	23.65%	31.50%
*Chronic patient*				
Nq500				
0 quarter	25.83%	29.62%	26.67%	31.32%
1 quarter	19.60%	21.37%	20.68%	22.41%
2 quarters	15.77%	16.73%	16.51%	16.11%
3 quarters	14.86%	14.29%	15.05%	13.68%
4 quarters	23.94%	17.98%	21.09%	16.49%
*Female*	60.43%	58.88%	59.81%	59.68%
*Cost ratio preceding year classes*				
1	4.63%	6.28%	4.44%	6.48%
2	4.68%	5.51%	4.79%	5.82%
3	4.69%	5.39%	4.92%	5.61%
4	4.71%	5.31%	4.84%	5.41%
5	4.71%	5.38%	5.04%	5.21%
6	4.72%	4.98%	5.03%	5.14%
7	4.73%	4.85%	5.07%	5.00%
8	4.73%	4.79%	5.27%	4.98%
9	4.75%	4.80%	4.84%	4.87%
10	4.75%	4.50%	4.89%	4.87%
11	4.76%	4.55%	4.91%	4.73%
12	4.77%	4.54%	4.86%	4.70%
13	4.79%	4.43%	4.56%	4.57%
14	4.79%	4.59%	4.74%	4.40%
15	4.80%	4.55%	4.69%	4.26%
16	4.80%	4.39%	4.64%	4.42%
17	4.82%	4.28%	4.47%	4.17%
18	4.83%	3.97%	4.40%	4.15%
19	4.83%	4.48%	4.59%	3.95%
20	4.84%	4.26%	4.65%	3.81%
21	1.21%	1.02%	1.25%	0.88%
22	1.22%	1.04%	1.04%	0.89%
23	1.22%	1.01%	1.03%	0.90%
24	1.23%	1.06%	1.05%	0.76%

**Table 2.** Econometric results

Variable	Mixed-effects model Coefficient [95% CI]	Std. Err.	Effect in per cent^a^
Hospital stay (HOSP)	0.959 [0.955; 0.962]***	0.002	160.8%
Nursing home stay (NURS)	0.241 [0.233; 0.250]***	0.004	27.3%
Insurance plan (PLAN)			
Capitated model (CAP)	–0.239 [–0.257; –0.221]***	0.009	–21.2%
Family doctor model (FDM)	–0.169 [–0.182; –0.157]***	0.006	–15.5%
Telemedicine model (TEL)	–0.038 [–0.046; –0.029]***	0.004	–3.7%
Cost ratio preceding year (CRPY)			
Class 2	–0.108 [–0.114; –0.101]***	0.003	–10.2%
Class 3	–0.126 [–0.134; –0.120]***	0.003	–11.9%
Class 4	–0.122 [–0.129; –0.115]***	0.003	–11.5%
Class 5	–0.110 [–0.117; –0.103]***	0.003	–10.4%
Class 6	–0.101 [–0.108; –0.094]***	0.003	–9.6%
Class 7	–0.090 [–0.098; –0.083]***	0.003	–8.7%
Class 8	–0.082 [–0.089; –0.076]***	0.003	–7.9%
Class 9	–0.068 [–0.075; –0.061]***	0.003	–6.5%
Class 10	–0.058 [–0.065; –0.051]***	0.004	–5.6%
Class 11	–0.039 [–0.046; –0.033]***	0.004	–3.9%
Class 12	–0.033 [–0.040; –0.026]***	0.004	–3.2%
Class 13	–0.017 [–0.024; –0.010]***	0.004	–1.7%
Class 14	–0.004 [–0.011; 0.003]	0.004	–0.4%
Class 15	0.018 [0.011; 0.025]***	0.004	1.8%
Class 16	0.043 [0.036; 0.050]***	0.004	4.4%
Class 17	0.064 [0.056; 0.070]***	0.004	6.6%
Class 18	0.094 [0.087; 0.101]***	0.004	9.9%
Class 19	0.131 [0.124; 0.138]***	0.004	14.0%
Class 20	0.208 [0.200; 0.214]***	0.004	23.1%
Class 21	0.300 [0.289; 0.310]***	0.006	35.0%
Class 22	0.382 [0.371; 0.393]***	0.006	46.5%
Class 23	0.513 [0.501; 0.524]***	0.006	67.0%
Class 24	0.786 [0.773; 0.798]***	0.006	119.4%
Accident coverage (ACC)	0.219 [0.215; 0.223]***	0.002	24.5%
Chronic patients (CHRO)			
1 Quarter (CHRO1)	1.121 [1.118; 1.125]***	0.002	206.9%
2 Quarters (CHRO2)	1.562 [1.558; 1.565]***	0.002	376.8%
3 Quarters (CHRO3)	1.861 [1.857; 1.865]***	0.002	542.8%
4 Quarters (CHRO4)	2.150 [2.156; 2.154]***	0.002	758.4%
Deductible class (DED)	–0.151 [–0.157; –0.145]***	0.003	–14%
Constant	–1.845 [–1.852; –1.839]***	0.003	–84.2%
Random effects Std. Err.			
Between group error	0.6075		
Within group error	0.5651		

*p<0.10, **p<0.05, ***p<0.01 ^a)^The regression analysis determines the regression coefficient β of each independent variable. In order to obtain the impact in percent of the dummy variables, the following further transformation of the coefficients is applied exp(*β*) – 1

**Table 3.** Calculation of the different effects by insurance model

Effect	A. Contracted models with capitation	B1. Contracted models without capitation	3B2. Telemedicine models
Overall average cost savings	–29.7%	–21.1%	–22.5%
There from efficiency effects	–21.2%	–15.5%	–3.7%
There from selection effects	–8.5%	–5.6%	–18.8%

**Table 4.** Comparison of cost ratios per pharmacy cost group by insurance model

Pharmacy cost group	Basic compulsory insurance model (Cost ratio mean)	A. Contracted models with capitation (Cost ratio mean and difference in percent from basic insurance)	B1. Contracted models without capitation (Cost ratio mean and difference in percent from basic insurance)	B2. Telemedicine models (Cost ratio mean and difference in percent from basic insurance)
PCG1	2.010	1.383	1.630	1.761
Asthma/COPD		–31.2%	–18.9%	–12.4%
PCG4	2.030	1.508	1.652	1.884
Cardiac disorders		–25.7%	–18.6%	–7.2%
PCG6	2.397	1.695	1.979	2.087
Gastric disorders		–29.3%	–17.4%	–12.9%
PCG11	1.675	1.224	1.467	1.427
Diabetes type II		–27.0%	–12.5%	–14.8%

## Footnotes

1 US$ purchasing power parity; the equivalent in Swiss Francs is 7546.

2 The concept of integrated care is in Switzerland a synonym for the well-known term of managed care.

3 The medical benefits covered by compulsory health insurance are defined by the federal government and are independent of the chosen health plan option.

4 There are no current official figures on the insured for a further model distinction. For this reason, in order to achieve an approximation an internal ratio of a major health insurance/sickness fund was used from the year 2009.

5 Residual value according to the previously calculated proportions (37% – 4% – 21.4%=11.6%; total ICM—capitated ICM—non-capitated ICM=non-contracted model).

6 The average costs utilised in the Swiss risk equalisation scheme are published annually and can be consulted on http://www.kvg.org/.

7 Asthma/chronic obstructive pulmonary disease (COPD), epilepsy, rheumatism, cardiac disorders, Crohn's disease/ulcerative colitis, gastric disorders, diabetes types I and II, Parkinson's disease, transplants, cancer, HIV/AIDS and kidney disorders.

8 We refer only to the significant results and therefore ignore the physician networks with a statistically insignificant result.

9 Achermann R. Abrechnungseffekte bei der Umstellung von tiers garant zu tiers payant. [Accounting effects in the transition from tiers garant to tiers payant.] Zurich: Helsana; 2006. [in German].

## Reviewers

**Reynaldo Holder**, MD, Regional Advisor – Hospitals and Integrated Health Care Delivery Systems, Area of Health Systems based on Primary Health Care, PAHO/WHO, Washington DC, USA

**Eva Krczal**, MD, Business School, Donau University, Dr. Karl Dorrek Straße, 30A-3500 Krems, Austria

**Matthias Schwenkglenks**, PhD, MPH, Head of Research, Institute of Pharmaceutical Medicine (ECPM), University of Basel, Switzerland and Head of Medical Economics Unit, Institute of Social and Preventive Medicine, University of Zurich, Switzerland
